# Assessing ternary materials for fluoride-ion batteries

**DOI:** 10.1038/s41597-023-01954-1

**Published:** 2023-02-11

**Authors:** Don H. McTaggart, Jack D. Sundberg, Lauren M. McRae, Scott C. Warren

**Affiliations:** Department of Chemistry, The University of North Carolina at Chapel Hill, Chapel Hill, NC 27599 USA

**Keywords:** Batteries, Computational methods, Batteries

## Abstract

Although lithium-ion batteries have transformed energy storage, there is a need to develop battery technologies with improved performance. Fluoride-ion batteries (FIBs) may be promising alternatives in part due to their high theoretical energy density and natural elemental abundance. However, electrode materials for FIBs, particularly cathodes, have not been systematically evaluated, limiting rapid progress. Here, we evaluate ternary fluorides from the Materials Project crystal structure database to identify promising cathode materials for FIBs. Structures are further assessed based on stability and whether fluorination/defluorination occurs without unwanted disproportionation reactions. Properties are presented for pairs of fluorinated/defluorinated materials including theoretical energy densities, cost approximations, and bandgaps. We aim to supply a dataset for extracting property and structural trends of ternary fluoride materials that may aid in the discovery of next-generation battery materials.

## Background & Summary

As our global system transitions to renewable energy, the demand for efficient and affordable energy storage will continue to grow. Currently, lithium-ion batteries (LIBs) bear the major load of electrochemical energy storage requirements due to their high energy density and cyclability^1^. However, it remains unclear whether the production of lithium-ion batteries can scale at a rate and cost that will meet future needs^2,3^. In the search for alternatives to lithium, fluoride-ion batteries (FIBs) are promising based on several factors. First, they offer higher theoretical energy densities than current LIBs (~1,000–2,200 Wh/kg vs 220–650 Wh/kg)^2,4^. Second, since fluorine is the most electronegative element, fluoride has high redox stability, which enables battery operation within a large electrochemical window. Third, fluoride and many of its prospective electrode materials are more abundant and less expensive than those for lithium^4^.

Significant work has been accomplished in discovering liquid electrolytes^5–8^ and high-performance anodes^9,10^ for FIBs. Although additional investigation in these components is still critical, there is an especially strong need to identify promising cathode materials. To guide previous cathode discovery, the primary heuristic that has been used is fluoride affinity. During the FIB discharge process, fluoride ions (F^−^) spontaneously leave the cathode and migrate through the electrolyte to the anode. For this to be spontaneous, the anode must have higher affinity for fluoride than the cathode. Thus, a general design rule for fluoride electrodes is that more electronegative elements such as Cu or Bi are promising for cathodes, and more electropositive elements such as Mg or Y are promising for anodes^11–15^. These simple guidelines have inspired considerable cathode work using the binary metal fluorides like Cu/CuF_2_, Fe/FeF_3_, Sn/SnF_2_ and particularly Bi/BiF_3_^12,16–20^. However, the realization of more complex compositions creates an opportunity to alter a material’s physical and electronic properties for cathode use beyond the limited scope of binary compositions. Recent studies have employed three and four elements-containing oxide cathode materials (La_2_NiO_4_, LaSrMnO_4_, La_2_CoO_4_, BaFeO_2.5_)^14,21–23^ to begin expanding this design space. Despite these advances, only a small number of more complex compositions have been investigated. In this work, we describe the first systematic exploration of compounds that assesses the cathodic capability of all known ternary fluoride materials.

**Fig. 1:**
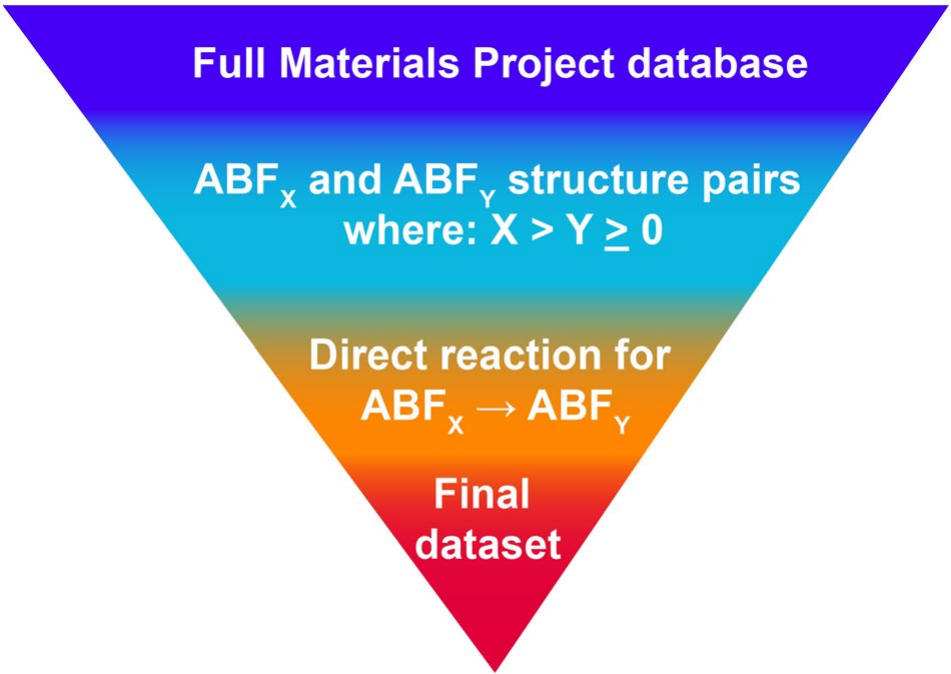
The filtering steps used to obtain the final dataset. Several quaternary materials were also identified as promising cathodes and included in this dataset.

With thousands of possible ternary metal fluorides, a method for selecting candidates for experimental discovery becomes paramount. The recent development of large crystal structure databases like Materials Project^24^ makes computational screening an attractive first step for this purpose. Here, we utilize computational filtering techniques to search for existing ternary metal fluorides in the Materials Project database that may function as cathodes for FIBs. Our search aims to identify fluorinated and defluorinated structure pairs. Identification of fluorinated/defluorinated pairs yields a dataset of possible charged and discharged states for a cathode material. This broader filtering is broken down into two smaller searches: fully defluorinated candidates and partially defluorinated candidates. These are further narrowed down by considering calculated stability and presence of disproportionation reactions along the defluorination pathway. In Fig. 1, we present a simplified diagram for this filtering algorithm.

**Fig. 2:**
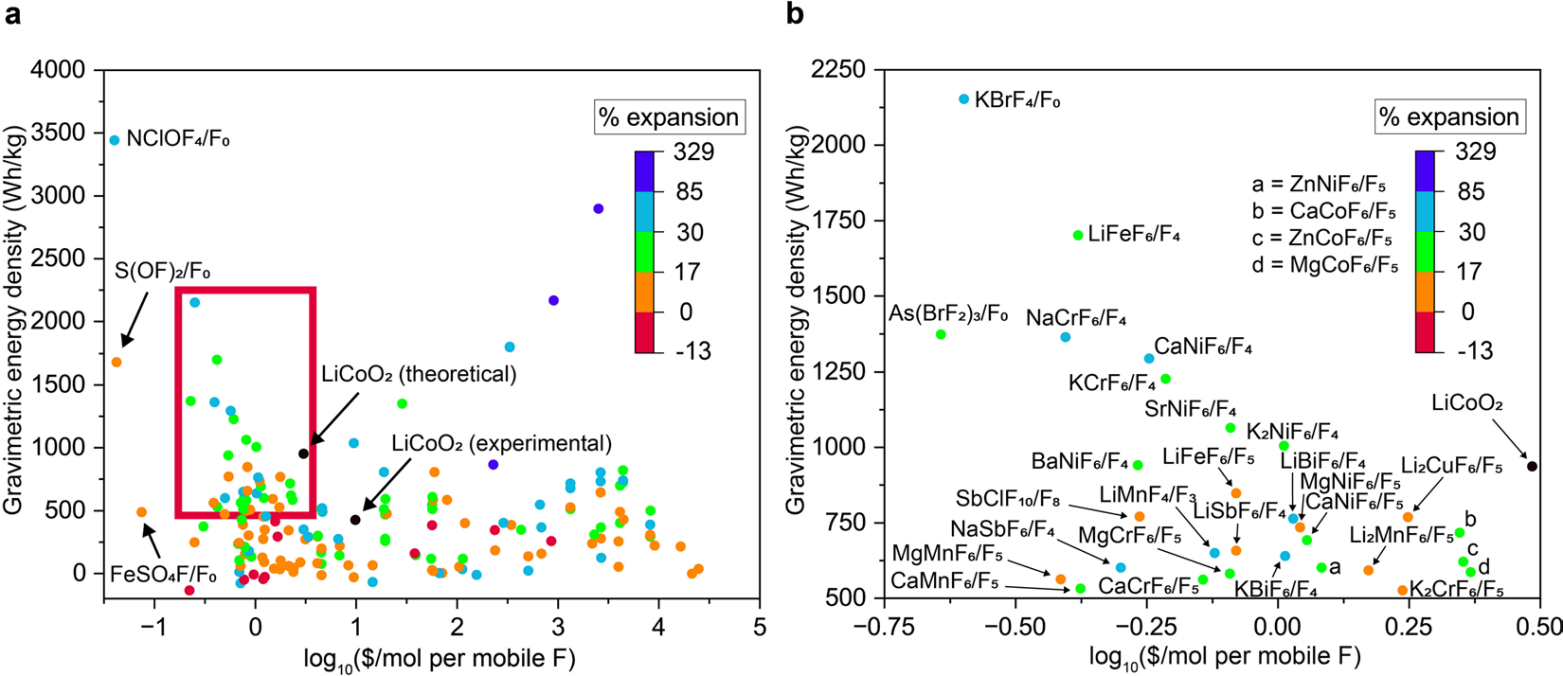
Cathodes demonstrating a direct reaction between fluorinated/defluorinated pairs. All 168 pairs are shown in (**a**). In (**b**) the graph is zoomed in to highlight the high energy density and low-cost materials. Each pair is identified by the fluorinated structure and “F_x_” which indicates the fluoride content of the defluorinated structure. The volumetric expansion of each pair is indicated by the color scale. Values of LiCoO_2_ (LIB cathode) are also included for comparison.

A collection of properties was also calculated for each structure pair including redox potentials vs. Li/LiF and vs. F_2_, specific capacity, percent expansion between defluorinated and fluorinated forms, energy densities, and cost approximations. Redox potentials vs. Li/LiF for direct reaction pairs ranged from −0.92 V (Ca_5_(PO_4_)_3_F/Ca_5_(PO_4_)_3_) to 5.77 V (LiAgF_6_/ LiAgF_4_) with an average of 2.9 V. Specific capacity ranged from 6.4 mAh/g (RbSnF_3_/RbSn_2.94_) to 758 mAh/g (NClOF_4_/NClO) with an average of 150 mAh/g. Figure 2a,b illustrate the relationship between energy density (Wh/kg) and log_10_($/mol of mobile F) with a colorimetric scale based on percent expansion for each pair. Log_10_($/mol of mobile F) is used rather than $/mol to visually counteract the Pareto-like distribution of low-cost materials and account for charge transfer capacity. Structures are distributed along the cost axis with a range that spans five orders of magnitude. Most structures have energy densities between 0–900 Wh/kg with moderate expansion between 7–40%. Figure 2b is a zoomed in view of **2a** at the desired property overlap of high energy density and low price with points identified by the fluorinated structure and fluoride content of the defluorinated structure.

Some fluorinated structures appear more than once with different defluorinated structures, such as CaNiF_6_/CaNiF_5_ and CaNiF_6_/CaNiF_4_. The presence of these multi-redox capable fluorinated structures highlights differences in chemical potential along a given defluorination pathway. When these materials are present, they are likely to also exhibit a multistep voltage profile. This is observed in the Ca-Ni-F system, where CaNiF_6_/CaNiF_5_ is 5.50 V vs. Li/LiF, CaNiF_5_/CaNiF_4_ is 4.78 V, and CaNiF_6_/CaNiF_4_ is 5.14 V. Although the two-electron transfer of CaNiF_6_/CaNiF_4_ pair is likely kinetically complicated compared to the single-electron transfer of the two former pairs, the variations in calculated potentials appear systematic. This type of consideration is expected to play an important role in cathode performance. Our dataset allows for straightforward identification of these and other types of interesting trends.

Structural factors must also be considered in assessing the viability of candidates. Although the NClOF_4_/NClO, S(OF)_2_/SO_2_, and As(BrF_2_)_3_/AsBr_3_ pairs shown in **2b** may seem promising, these materials are molecular crystals. These crystals likely lack the conductivity and mechanical stability needed to function as an electrode. Thus, these graphs provide only a snapshot of some properties and should not be taken as the only important criteria. Metrics for the common LIB cathode LiCoO_2_ are also displayed on the graph to orient the reader. The theoretical energy density for LiCoO_2_ is calculated in the same manner as the energy densities for the fluorination pairs, while the experimental energy density for LiCoO_2_ references current literature values^3^. The discrepancy in energy density between the theoretical and experimental values of LiCoO_2_ has been attributed to the incomplete deintercalation of Li from LiCoO_2_, which is also accounted for in the cost calculation^25^.

Although our dataset may encourage researchers to focus on materials with energy densities above those of current LIB technology, we also note that other characteristics can be important. These include ionic and electrical conductivity, chemical compatibility with an electrolyte, electrochemical stability, and material recyclability. Some of these additional criteria are also included in our dataset and we encourage researchers to evaluate the dataset holistically. On a broader scale, we hope that this dataset can be used to guide experimental selection of promising new FIB cathodes.

## Methods

The dataset was produced using the Simulated Materials Ecosystem (Simmate)^26^ and Materials Project API^24^. Simmate combines many crystal structure databases in one software package, including Materials Project, the Crystallography Open Database (COD)^27^, the Joint Automated Repository for Various Integrated Simulations (JARVIS)^28^, and the Open Quantum Materials Database (OQMD)^29^. At the time of writing, the Materials Project database contains over 145,000 crystal structures. The Materials Project database (via Simmate) was initially filtered to include all fluoride-containing structures with more than two elements and a hull energy less than or equal to 75 meV (6,525 structures). This cut-off was chosen based on known errors in DFT formation enthalpies and is discussed further in Technical Validation. To ensure that each composition was represented solely by the most stable phase, these structures were sorted by ascending hull energy and entries after the first occurrence of a given reduced formula were excluded. This list comprised the fluorinated half of all possible fluorinated/defluorinated pairs (4,389 structures).

### Complete defluorination

A copy of each fluorinated entry was made, and all fluorine atoms were removed from the copied structure. This represented the other half of a possible fluorinated/defluorinated pair. The reduced formula of the defluorinated copy was then compared to the full Materials Project database. If an entry on Materials Project had the same reduced formula and was within 75 meV of hull, the entry was identified as a possible match. Multiple phases of the same composition also appeared during this step and were addressed in a similar manner as above to identify the most stable phase. There were 425 fluorinated/fully defluorinated pairs identified after this step.

### Partial defluorination

Identification of partially defluorinated structure pairs was more involved than complete defluorination, as structural compositions along the full defluorination pathway had to be considered. The Materials Project database was searched for each entry in the initial fluorinated list, where structures with the same elements as the fluorinated entry, with the same ratios of non-fluoride elements, and within 75 meV of hull, were identified as possible defluorinated pairs. For structures with identical compositions, we filtered the dataset to select the lowest energy phase. This resulted in 382 fluorinated/partially defluorinated pairs. Thus, a total of 807 fluorinated/defluorinated pairs were taken forward through the remaining analyses.

### Disproportionation reactions

**Fig. 3:**
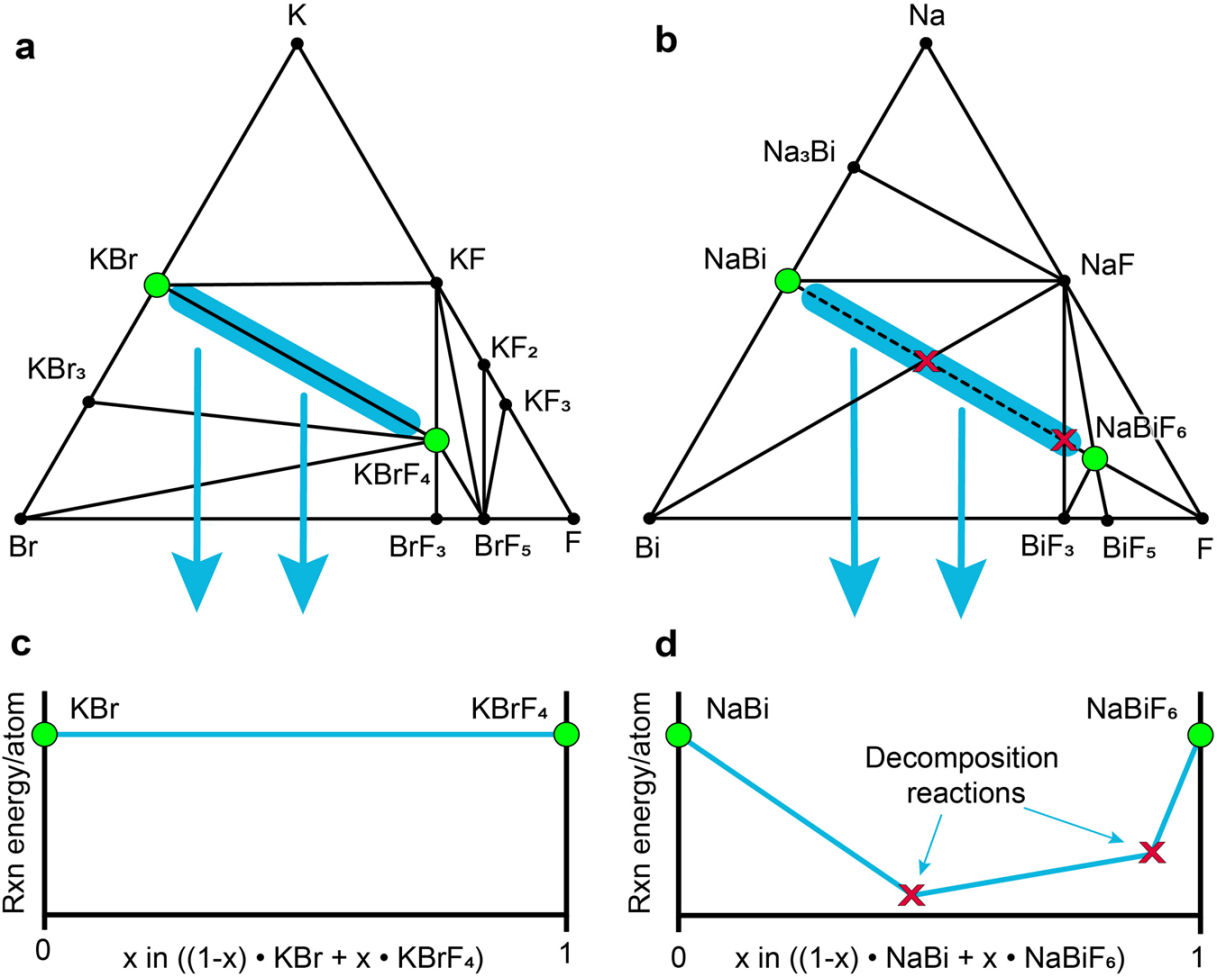
Examples of (**a,c**) direct and (**b,d**) indirect interface reactions for fluorinated/defluorinated structure pairs. In (**a**) there is a single tie-line connecting the fluorinated (KBrF_4_) and defluorinated (KBr) structures. In (**b**) the drawn-in dotted-line between NaBiF_6_ and NaBi crosses over the tie-lines connecting NaF/BiF_3_ and NaF/Bi, leading to disproportionation to the phases on either end of the crossed tie-lines as F is removed. The interface reaction coordinates (c/d) reflect these predictions.

The ternary phase diagram of a three-element system is characterized by nodes and tie-lines. Nodes are specific stable (or metastable) structures and tie-lines represent two-phase equilibria between these structures^30^. When a fluorinated/defluorinated pair is identified, it becomes imperative to check the associated phase diagram for a tie-line connecting the two, which shows that there is a direct reaction between the two phases. In the absence of a direct reaction, the fluorinated/defluorinated pair will disproportionate into two or more additional phases at global compositions that are intermediate between the two pairs. To illustrate these direct and indirect reactions, in Fig. 3 we show the phase diagrams and reaction coordinates for two possible fluorinated/defluorinated systems. It is useful to interpret the tie-line (**3a**) and dotted-line (**3b**) connecting the two fluorinated/defluorinated structures on the phase diagram as top-down views of reaction coordinates. Each diagram and reaction coordinate shows an example of a direct (**3a,c**, KBrF_4_/KBr) and indirect (**3b,d**, NaBiF_6_/NaBi) reaction. In **3a** the KBrF_4_/KBr pair has a tie-line between the two target structures (circled in green), indicating that the structures convert from one to the other as fluorine content changes via a direct reaction. This is reflected in the interface reaction coordinate (**3c**) with a straight, horizontal line indicating that the pair does not decompose into other phases. However, there is no tie-line connecting the NaBiF_6_/NaBi pair (**3b**, dotted line drawn in to highlight where a tie-line would exist). In fact, there are two other tie-lines that lie between the pair. This means that at global compositions between NaBiF_6_ and NaBi, the system will at least partially disproportionate into NaF, BiF_3_, and Bi. The interface reaction coordinate (**3d**) confirms that these disproportionation reactions are thermodynamically favourable, indicated by the decrease in reaction energy per atom. Once disproportionation occurs, the presence of multiple heterogeneous phases makes it less likely to form the desired product (eg NaBi), even if the global composition exactly matches NaBi. In this event, the amount of active material and the cathode’s cyclability will decrease.

To assess each fluorinated/defluorinated pair for the presence of direct or indirect reactions, we used pymatgen’s interface reaction function^31^. This function used the fluorinated and defluorinated structure pair as the two reactants. If there was no thermodynamically favorable reaction between the pair, the function gave the two original structures as “products” of the reaction. This indicated a direct reaction. If an indirect (disproportionation) reaction occurred, the function returned a list of disproportionation products. Of the 807 pairs, 168 had direct reactions. The first 58 of these were complete defluorination pairs, which was 14% of the original 425 complete defluorination pairs. The remaining 110 direct reactions were partial defluorination pairs, or 29% of the original 382 partial defluorination pairs.

Relevant properties were calculated for the 168 direct reaction structure pairs:

### Potentials

It is known that open circuit voltages can be predicted for lithium-ion battery materials using energies calculated from DFT + U methods^32^. It is common to refer to voltages against metallic lithium in these systems, which can be considered the natural limit for the usability of a component in a lithium-ion battery. In fluoride-ion batteries, F_2_ can be used analogously to calculate open circuit voltages for materials in these systems^23^. These potentials can be calculated for materials by using a hypothetical F_2_ electrode according to the following reaction:

1 $${\rm{AB}}+\frac{x}{2}{{\rm{F}}}_{2}\to {{\rm{ABF}}}_{{\rm{x}}}$$

While this reaction is useful theoretically, the realization of a gaseous F_2_ electrode is unlikely. Moreover, as written, the potentials obtained are oxidation potentials rather than reduction potentials. To provide a more conventional perspective, we also calculate potentials using the Li/LiF pair as an anode according to:

2 $${{\rm{ABF}}}_{{\rm{x}}}+{\rm{x}}\,{\rm{Li}}\to {\rm{AB}}+{\rm{x}}\,{\rm{LiF}}$$

For this reaction, a good cathode material corresponds to a high voltage. The voltages for these two reactions can be calculated starting from the Nernst equation:

3 $$Potential=-\frac{\Delta {G}_{{\rm{rxn}}}}{xF}$$

Where ΔG_rxn_ is the change in Gibbs free energy of the reaction (J/mol), *x* is the number of fluoride ions transferred, and *F* is Faraday’s constant. Volume (PΔV_rxn_) and entropic (TΔS_rxn_) effects can be neglected for the calculation of ΔG_rxn_ (=ΔU_rxn_ + PΔV_rxn_ – TΔS_rxn_) because PΔV_rxn_ is on the order of 10^−5^ eV and TΔS_rxn_ is on the scale of thermal energy, which are both much smaller than ΔU_rxn_ (~10^1^ eV). Thus, ΔG_rxn_ can be reasonably obtained from the change in internal energy (ΔU_rxn_)^33–35^. The Materials Project calculates internal energy for every entry in its database using DFT + U, so ΔG_rxn_ can be calculated from these values for (1) and (2) respectively according to:

4 $$\Delta {{\rm{G}}}_{{\rm{rxn}}}\approx \Delta {{\rm{U}}}_{{\rm{rxn}}}=({{\rm{U}}}_{{\rm{ABFx}}})-\left({{\rm{U}}}_{{\rm{AB}}}+\frac{x}{2}{{\rm{U}}}_{{\rm{F}}2}\right)$$

and

5 $$\Delta {{\rm{G}}}_{{\rm{rxn}}}\approx \Delta {{\rm{U}}}_{{\rm{rxn}}}=\left({{\rm{U}}}_{{\rm{AB}}}+{{\rm{xU}}}_{{\rm{LiF}}}\right)-\left({{\rm{U}}}_{{\rm{ABFx}}}+{\rm{x}}\,{{\rm{U}}}_{{\rm{Li}}}\right)$$

where U_y_ is the internal energy of each compound. Because Materials Project provides internal energy values in eV rather than J, Faraday’s constant can be dropped from (3). Thus, potentials vs. F_2_ and vs. Li/LiF were calculated from -ΔG_rxn_/x. These oxidation potentials range from −0.35 to 7.07 V vs. F_2_ and the reduction potential range from −0.92 to 6.50 V vs. Li/LiF. We use the potentials vs. Li/LiF for energy density calculations. Graphite and LiC_6_ were used instead of Li and LiF to calculate the potential for LiCoO_2_/CoO_2_ (LIB cathode).

### Volume per fluorine (Å^3^)

Volume per fluoride was calculated using the volume per formula unit of the fluorinated structure divided by the difference in fluorine atoms between the fluorinated and defluorinated structures.

### Percent expansion

The expansion from the defluorinated to fluorinated structure was calculated by dividing the fluorinated volume per formula unit by the defluorinated volume per formula unit, subtracting 1, and then multiplying by 100%.

### Gravimetric capacity (mAh/g)

The gravimetric capacity for each fluorinated structure was calculated according to Faraday’s Law:

$$Capacity=\frac{n\ast F}{C\ast MW}$$

Where *n* is the number of charge carriers (fluoride ions), *F* is Faraday’s constant (96,485.3 C/mol), *C* is a conversion factor (3.6 C/mAh), and *MW* is the molecular weight of the fluorinated structure in g/mol.

### Gravimetric energy density (Wh/kg)

The gravimetric energy density was obtained by multiplying the gravimetric capacity by voltage.

### Volumetric energy density (Wh/L)

The volumetric energy density was calculated by multiplying the gravimetric energy density by the density of the fluorinated material.

### Cost analysis

A cost analysis was done for each material using pymatgen’s cost module. Costs were not calculated for Rb_2_UF_7_ due to issues with the module in calculating a phase diagram containing U. For readers interested in replicating these results, column headers must be deleted from the ‘costdb_elements_new.csv’ file before using for the analysis to work properly.

### Transport barrier (eV)

When data was available, the activation energy for fluoride transport is presented^36^.

The difference in atoms per formula unit between each structure pair, which is used to calculate the number of fluorides transferred and other properties, assumes that each reduced formula has the same number of each type of non-F atoms. This is not true for all structures, however. Therefore, we counted the number of non-fluoride atoms in each pair and rescaled the reduced formulas so that the number of non-fluoride atoms remained unchanged.

Several 4- and 5-element containing pairs were also identified including Ca_5_(PO_4_)_3_F, Ca_5_(VO_4_)_3_F, Pb_5_(PO_4_)_3_F, Pb_5_(VO_4_)_3_F, LiVPO_4_F, LiMnPO_4_F, LiFePO_4_F, CeAsO_4_F, BaAlGeF, TmSeO_3_F, LuSeO_3_F, LiCrPO_4_F, Sr_2_FeO_3_F, Ca_6_Al_3_(AlO_4_)_4_F, Na_3_MoO_4_F, YSeO_3_F, Li_2_CoO_2_F, TiH_8_(NF_3_)_2_, ReSbOF_10_, MgAs_2_(XeF_9_)_2_. The filtering criteria worked correctly for these structures and were left in as additional datapoints.

## Data Records

The produced datasets are available in Figshare^37^ and at the ternary_f_cathodes page on Github. The datasets are provided as two CSV files, one containing all 807 pairs and one containing the 168 direct reaction pairs. The modified cost spreadsheet is also provided. The python script file used to create the dataset is available on the Github page. Table 1 provides the list of column titles in the files and a description of each.

**Table 1:** List of column titles in cathode datasets with descriptions.

Column name	Description
orig_index	Original index from initial database filtering
f_form	Reduced formula of fluorinated structure
def_form	Reduced formula of defluorinated structure
voltage_lif	Voltage of F/deF pair vs. Li/LiF
voltage_f2	Voltage of F/deF pair vs F_2_
vol_p_f	Volume per mobile F (Å^3^) (fluorinated)
per_exp	Percent expansion from defluorinated to fluorinated
grav_cap	Gravimetric capacity (mAh/g) (fluorinated)
grav_e_den	Gravimetric energy density (Wh/kg) (fluorinated)
vol_e_den	Volumetric energy density (Wh/L) (fluorinated)
f_transfer	Number of mobile F ions between pair
f_cost_p_kg	$/kg of fluorinated structure
def_cost_p_kg	$/kg of defluorinated structure
f_cost_p_mol	$/mol of fluorinated structure
def_cost_p_mol	$/mol of defluorinated structure
cost_p_mol_f	$/mol of mobile F ion (fluorinated)
log_cost_p_mol_f	Log of $/mol of mobile F ion (fluorinated)
f_id	Materials Project ID (fluorinated)
def_id	Materials Project ID (defluorinated)
f_spacegroup	Spacegroup number (fluorinated)
def_spacegroup	Spacegroup number (defluorinated)
f_e_hull	Hull energy (eV) (fluorinated)
def_e_hull	Hull energy (eV) (defluorinated)
f_bandgap	Bandgap (eV) (fluorinated)
def_bandgap	Bandgap (eV) (defluorinated)
f_barrier	Diffusion barrier (eV) (fluorinated)
def_barrier	Diffusion barrier (eV) (defluorinated)
direct	Is conversion a direct reaction?
products	Products of interface reaction (equal to F structure if direct)
type	Complete or partial defluorination

## Technical Validation

The data presented is taken from Simmate and Materials Project databases or calculated directly according to the methods described above. Many of the reported properties rely on DFT internal energy calculations, which are known to incorporate systematic and random error. Particularly, the approximate DFT functionals result in errors due to changes of electron self-interaction in localized transition metal states. This originates from changes in oxidation state upon product formation from discrete reactants. Known binding errors of diatomic molecules (F_2_, O_2_, N_2_, Cl_2_, H_2_) using LDA and GGA functionals further complicate the calculation of accurate internal energies. This is mitigated in transition metal cations by applying a Hubbard U value to d or f orbitals and constant energy corrections based on experimental comparison for anionic species (F^−^, O^2−^, S^2−^…). Materials Project employs corrections for both errors with a mix of GGA and GGA + U functionals as well as anion-specific corrections^38–42^. Our calculations used these corrections, which reduces the error in GGA or GGA + U formation enthalpies from ≈ 175–450 meV/atom (uncorrected) to ≈ 45–55 meV/atom (corrected). The magnitude of these energies supported the chosen filtering value of 75 meV above hull, slightly above the noted correction error. The calculation of cathode potential relies on internal energies of multiple structures, which somewhat increases the total error. These errors are small compared to the potentials that are spanned across all cathodes, thereby supporting our ability to sort and rank cathode according to the predicted potential. The uncertainties of activation energies for F-ion conduction were described previously^36^.

Adaptations were made to pymatgen’s cost module for more accurate price approximations. Original module reference prices are based on pure elemental forms, which misrepresent the predicted costs because many elements can be obtained as salts for a much lower cost. Therefore, all elemental costs are obtained from the Wikipedia “Prices of chemical elements” page, which more accurately takes this into account. The prices listed on Wikipedia are primarily average market prices for bulk trade, but when this data is not available the price of a compound is used, per mass of the given element.

## Data Availability

All code used is open source and available at https://github.com/donmctaggart15/ternary_f_cathodes. The datasets are provided on the same repository. We recommend reading the Simmate, Materials Project API, and pymatgen documentation to follow filtering syntax.
